# A double-blinded randomised controlled trial – do subcuticular non-absorbable skin sutures have a better aesthetic outcome than skin staples in large wound closures?

**DOI:** 10.3205/iprs000142

**Published:** 2019-11-15

**Authors:** Joshua Agilinko, Poh Tan

**Affiliations:** 1 Burton Hospitals, NHS Foundation Trust, Staffordshire, United Kingdom

**Keywords:** skin suture, skin staple, wound closure

## Abstract

**Introduction:** Traditionally, non-absorbable skin sutures (SS) have been utilised in the closure of the skin in large wounds. More recently, however, skin staples (SC) have been introduced with the aim of reducing closure time and infection rates.

**Method:** A double-blinded randomized controlled trial in all patients undergoing elective open surgeries in a single unit, from May 2007 to May 2010. Data on patient demographics, type of surgery, methods of skin closure, rate of wound infection and cosmetic satisfaction were collected. Patients were then randomly allocated to skin sutures (SS) or skin staples (SC) groups. Patients and investigators were then “blinded” to the arm of trial they were allocated to.

**Result:** In total, 369 patients were recruited, of which 218 patients completed the study. 134 patients were allocated to the SS group with a median age of 67 (IQR 61, 74). SC group had a total of 84 patients with a median age of 69 (IQR 61, 71). 15% of SS group developed wound infection, compared to 20% in SC group (p=0.202). 61% of the SS group claimed better aesthetic results compared to 46% in SC group (p=0.020).

**Conclusion:** Our results demonstrated that patients with non-absorbable subcuticular skin closures had lower infection rates, better cosmetic outcome and better patient satisfaction outcome compared with skin staples. We therefore suggest using subcuticular sutures to close the skin in elective abdominal open surgery.

## Zusammenfassung

**Einführung:** Traditionell wurden nicht resorbierbare Hautnähte zum Verschließen der Haut bei großen Wunden verwendet. In jüngerer Zeit wurden jedoch Hautklammern eingeführt, um die Verschlusszeit und die Infektionsrate zu senken.

**Methode:** Eine doppelblinde, randomisierte, kontrollierte Studie mit allen Patienten, die von Mai 2007 bis Mai 2010 einer einzeitigen elektiven offenen Operation unterzogen wurden. Es wurden Daten zur Patientendemographie, Art der Operation, Methoden zum Schließen der Haut, Wundinfektionsrate und kosmetischen Zufriedenheit gesammelt. Die Patienten wurden dann nach dem Zufallsprinzip den Gruppen Hautnähte (SS) oder Hautklammern (SC) zugeordnet. Patienten und Ermittler wurden dann für den Arm der Studie, dem sie zugeteilt wurden, „verblindet“.

**Ergebnis:** Insgesamt wurden 369 Patienten rekrutiert, von denen 218 Patienten die Studie beendeten. 134 Patienten mit einem Durchschnittsalter von 67 Jahren wurden der SS-Gruppe zugeordnet (IQR 61, 74). Die SC-Gruppe hatte insgesamt 84 Patienten mit einem Durchschnittsalter von 69 Jahren (IQR 61, 71). 15% der SS-Gruppe entwickelten eine Wundinfektion, verglichen mit 20% in der SC-Gruppe (p=0,202). 61% der SS-Gruppe gaben bessere ästhetische Ergebnisse an, in der SC-Gruppe waren es nur 46% (p=0,020).

**Fazit:** Unsere Ergebnisse zeigten, dass Patienten mit nicht resorbierbaren subkutikulären Hautverschlüssen im Vergleich zu Hautklammern geringere Infektionsraten, ein besseres kosmetisches Ergebnis und ein besseres Ergebnis der Patientenzufriedenheit aufwiesen. Wir empfehlen daher die Verwendung von subkutikulären Nähten, um die Haut bei elektiven offenen Bauchoperationen zu schließen.

## Introduction

A good wound closure is one that has an excellent cosmetic result with minimal complications such as wound dehiscence and infection [1], [2].

In current practice, non-absorbable skin sutures (SS) and staples (SC) have been options used for large wound closure.

Whilst staples are regarded as quicker and easier to use than sutures [3], [4], [5], they produce poorer aesthetic outcomes and have been shown to be associated with a significantly greater risk of wound infection than traditional suturing [3].

Comparatively, studies have shown traditional suturing to have superior cosmetic results as well as lower infection rates [1], [2], [3], [4].

We aim to assess the difference between sutures and staples used in elective surgery in terms of wound infection and patient satisfaction.

## Methods and material

### Patients

This is a double-blinded randomized controlled trial including all the patients who underwent elective open surgery in our surgical department from May 2007 to May 2009. Patients were allocated randomly into two groups by the operating surgeon’s choice depending upon the method of abdominal wound closure. Group 1 included the patients who had abdominal skin closure using non-absorbable skin sutures (SS) and Group 2 included patients who had percutaneous staples for their skin closure (SC).

Both groups of patients were followed up using a Quality of Life Questionnaire to assess the scar. The questionnaire was designed based on European and local guidelines and approval was taken from the clinical governance department (Table 1 (Tab. 1)).

#### Inclusion criteria

1. Patients underwent elective open abdominal surgery
2. Patients with a virgin abdomen

#### Exclusion criteria

1. Previous laparotomy incision
2. Emergency open abdominal surgery

### Quality of life and patient satisfaction

A robust questionnaire Performa was used to assess the quality of life and patient satisfaction for abdominal skin closure after abdominal surgery (Table 1 (Tab. 1)). It enquired about scar, infection, pain and overall cosmesis outcome. Patients answered specific questions about infection (during their stay or after discharge from hospital), the use of antibiotics or the need for surgical management. Cosmetic results were assessed based on scar appearance, colour, thickness, width and numbness. Another aspect of the questionnaire was the management of any other scar problems in the community such as pain management and the use of gel or ointments. Wound infection was defined by purulent discharge, abscess formation or spreading cellulitis within the first month.

### Ethical approval

An ethical approval for the study was taken from the patient public department before the start of the trial. All patients were consented to the procedure at the time of operation.

### Data collection

369 patients were identified as having elective open surgery from May 2007 to May 2009. Data was collected on patient age, gender, type of procedure, comorbidities (ASA score), method of skin closure and patient questionnaire.

### Follow-up

All patients were seen at the outpatient clinic at 6-week and 3-month intervals for the first year after the surgery. All the patient questionnaires were collected at the second clinic visit.

### Statistical analysis

Statistical package for social science (SPSS) version 16.0 was used to perform statistical analyses of the available data. Pearson chi-square test was used to analyze the data. Mean and median values were compared by standard statistical tests as appropriate. A P value of less than 0.05 was considered significant.

## Results

369 patients were identified as having elective colorectal surgery from May 2007 to May 2009. 151 patients did not meet the inclusion criteria. A total of 218 filled questionnaires were received. 134 patients were allocated to the SS group with a median age of 67 (IQR 61, 74). The SC group had a total of 84 patients with a median age of 69 (IQR 61, 71).

### Infection

Infection rates were divided into two categories: infection as in-patient and overall infection rates. Only 7% of the SS group patients developed infection while in-patients, as compared to 15% of the SC group (p=0.033). But overall, 15% of the SS group developed wound infection, as compared to 20% in the SC group (p=0.202) (Table 2 (Tab. 2), Table 3 (Tab. 3), Table 4 (Tab. 4)).

### Scar appearance

17% of the SS group had unsatisfactory scar thickness as compared to 28% in the SC group (p=0.041), 30% in the SS group noticed a widened scar compared with 45% in the SC group (p=0.019). Only 38% of the SS group could feel the scar as compared with 51% of the SC group (p=0.040). The majority of patients in the SS group (59%) noticed that their scar matched the skin colour, as compared to 45% of the SC group (p=0.042).

There was no significant difference noticeable in prominence, swelling and tension of the scar in both the groups (Table 2 (Tab. 2), Table 3 (Tab. 3), Table 4 (Tab. 4)).

#### Satisfaction rates

78% of patients in the SS group were very satisfied with scar formation as compared to 55% of patients in the SC group (p=0.015). Overall, 61% of the SS group claimed excellent results compared to 46% in the SC group (p=0.020) (Table 2 (Tab. 2), Table 3 (Tab. 3), Table 4 (Tab. 4)).

#### Pain and analgesia

There was no significant difference noted in pain and tension of the scar in both the groups.

## Discussion

Surgical site infection (SSI) is classified by the World Health Organization (WHO) as any purulent discharge, abscess, or spreading cellulitis at the surgical site within a month of the operation.

With SSIs causing over one third of post-operative related deaths [6], pressure is placed on health care professionals and especially surgeons in preventing the spread of infections amongst hospitalized patients. Nosocomial infection carries a high mortality rate and causes a significant burden on morbidity with 1.4 million worldwide suffering from infectious complications acquired in hospitals [7].

Furthermore, SSI causes poor scars that are cosmetically unsatisfactory, such as those that are widened, hypertrophic or keloid, persistent pain and itching, restriction of movement, and it significantly impacts on emotional wellbeing [8].

Our study suggests that metallic staples caused more wound infection, with a significant number of patients developing infection during hospital stay (p=0.033). Interestingly, male patients developed more wound infections (p=0.05) particularly during hospital stay (p=0.042) (Table 2 (Tab. 2), Table 3 (Tab. 3), Table 4 (Tab. 4)).

Earlier studies suggested that a reduction in infection rates could be achieved with skin staples because staples do not penetrate the incision but cross the incision site [9] and might cause less damage to the wound’s defenses than non-absorbable sutures [9], [10]. However, a recent evidence suggested a higher risk of wound infection with the use of metallic staples than sutures and favors the use of sutures to close the wound [11].

In regard to cosmesis, our results showed that metallic staples increase the thickness (p=0.041) and width (p=0.019) of the scar as compared to non-absorbable subcuticle stitches (Table 4 (Tab. 4)). Similar results regarding thickness of the scar were reported by Bragg et al. [11] (Table 2 (Tab. 2), Table 3 (Tab. 3), Table 4 (Tab. 4)).

A significantly higher number of patients in the SC group claimed that the scar was palpable even after one year (p=0.040). Our results showed that patients in the SS group noticed that their scar matches the skin colour after one year (p=0.042). Male patients in this study were more satisfied with the color of the scar than the female patients. The reason may be the differing skin texture, in that male skin tends to have higher concentration of melanin and is more pliable than female skin. The differences allow better healing [12] (Table 2 (Tab. 2), Table 3 (Tab. 3), Table 4 (Tab. 4)).

We also noticed that there was no significant difference in prominence, swelling and tension of scar in both the groups.

Our results illustrate that the subcuticular sutures are superior to metallic staples in terms of cosmesis of the wound and patient satisfaction. Some studies suggested that poor results from staples are attributable to poor technique of staple placement. This leads to not only poor healing but also to wound discharge leading to wound infection [13], [14], [15]. Staples are considered to be more expensive, and the staple removal device poses extra burden to the cost [14], [15].

Furthermore, staples increase the rate of wound infection, which ultimately means an increased number of dressings and nursing costs. Finally, metallic staples are reported to be more painful than sutures [8], [15], [16], [17], [18]. Ranaboldo et al. also showed increase in pain and increase in the use of analgesia with metallic staples, which is associated with more distress and morbidity [19].

None of the studies have investigated the cost related to wound infection and further management in terms of antibiotic use, GP visits and further surgical interventions.

Patients with sutures were generally more satisfied with scar formation (p=0.015) and overall excellent (p=0.020) results compared to the staples. Lubowski et al. showed no difference in complications between the two techniques, but there was convincing evidence that staples resulted in poor cosmetic results [20], [21].

## Conclusions

Our results showed that patients with non-absorbable subcuticular skin closure compared to staples had reduced infection rates and were more satisfied with their scar and the cosmesis outcome. We therefore suggest using sub-cuticle sutures to close the skin in abdominal elective open surgery. We recommend larger randomised control trials in the future.

## Notes

### Competing interests

The authors declare that they have no competing interests.

## Figures and Tables

**Table 1:**
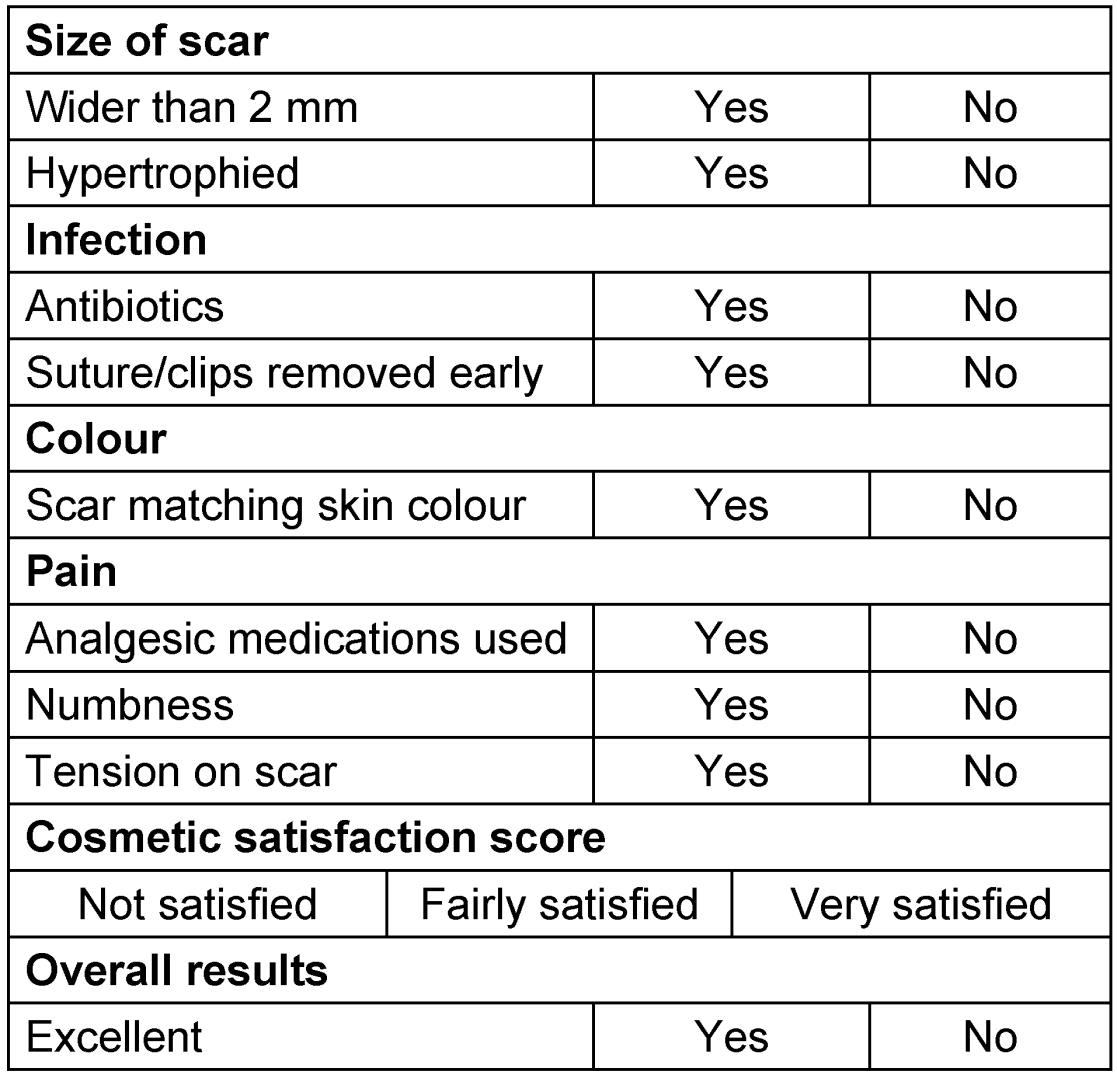
Quality of life and patient satisfaction proforma

**Table 2:**
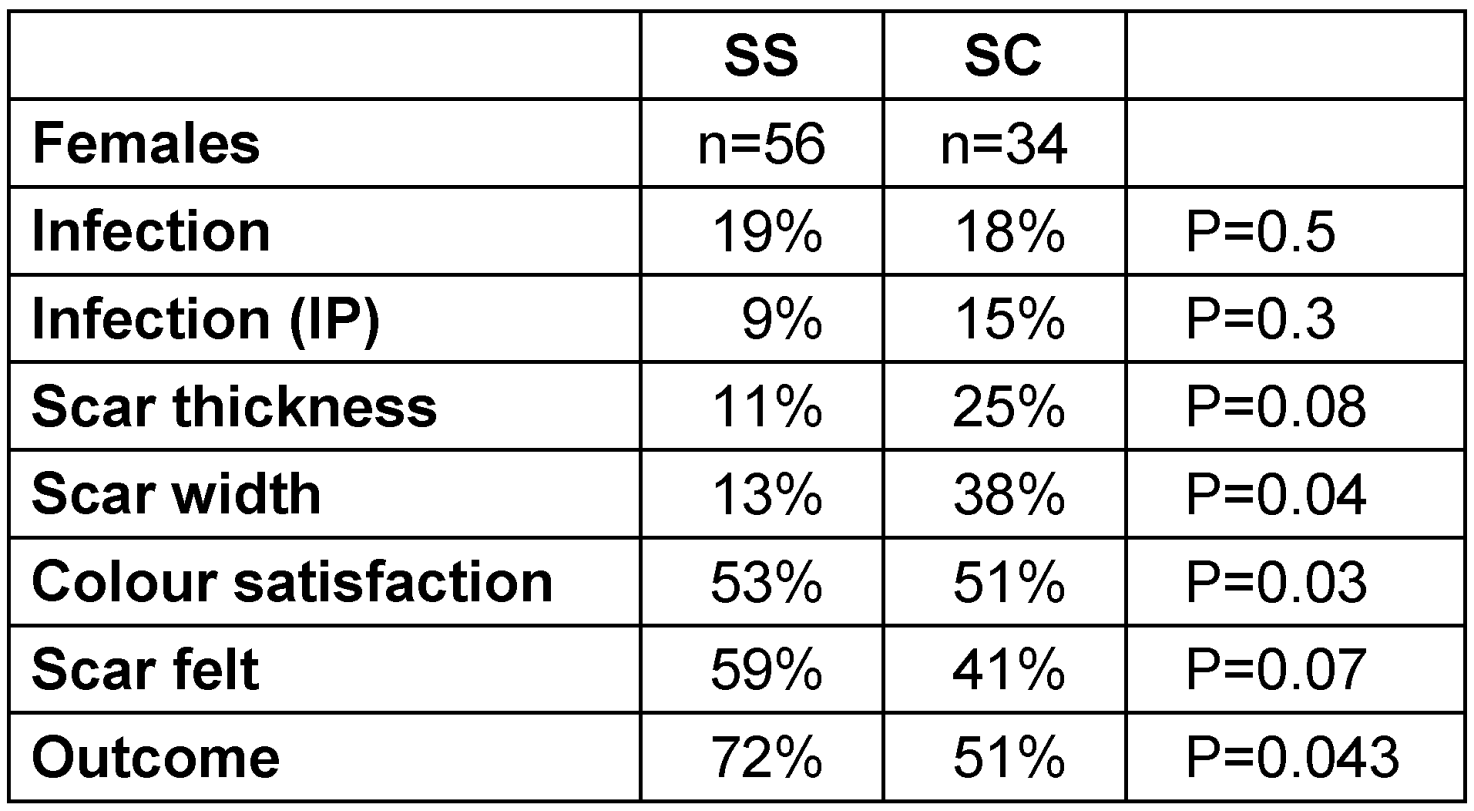
Overall result for female population of both SS and SC groups

**Table 3:**
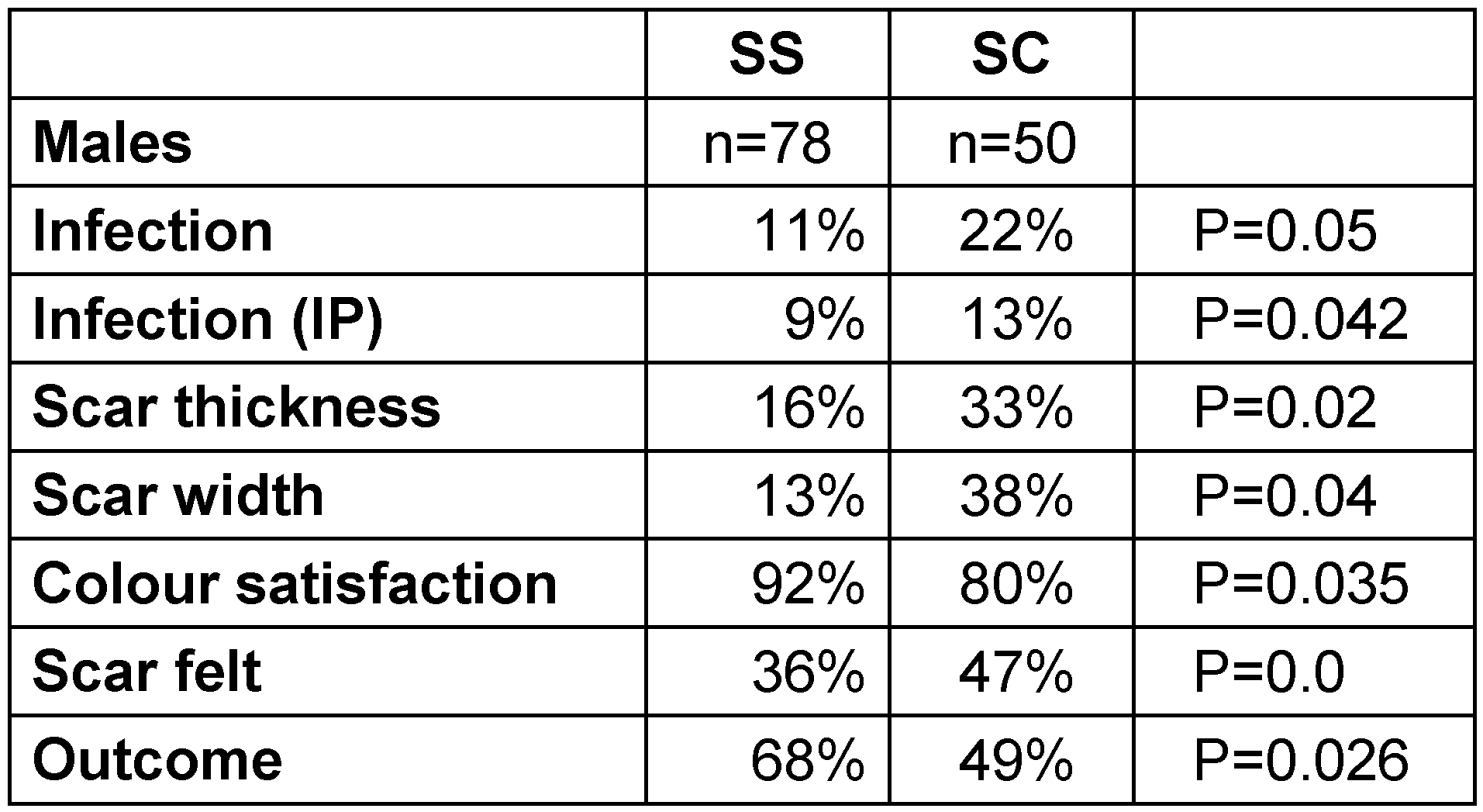
Overall result for male population of both the SS and SC groups

**Table 4:**
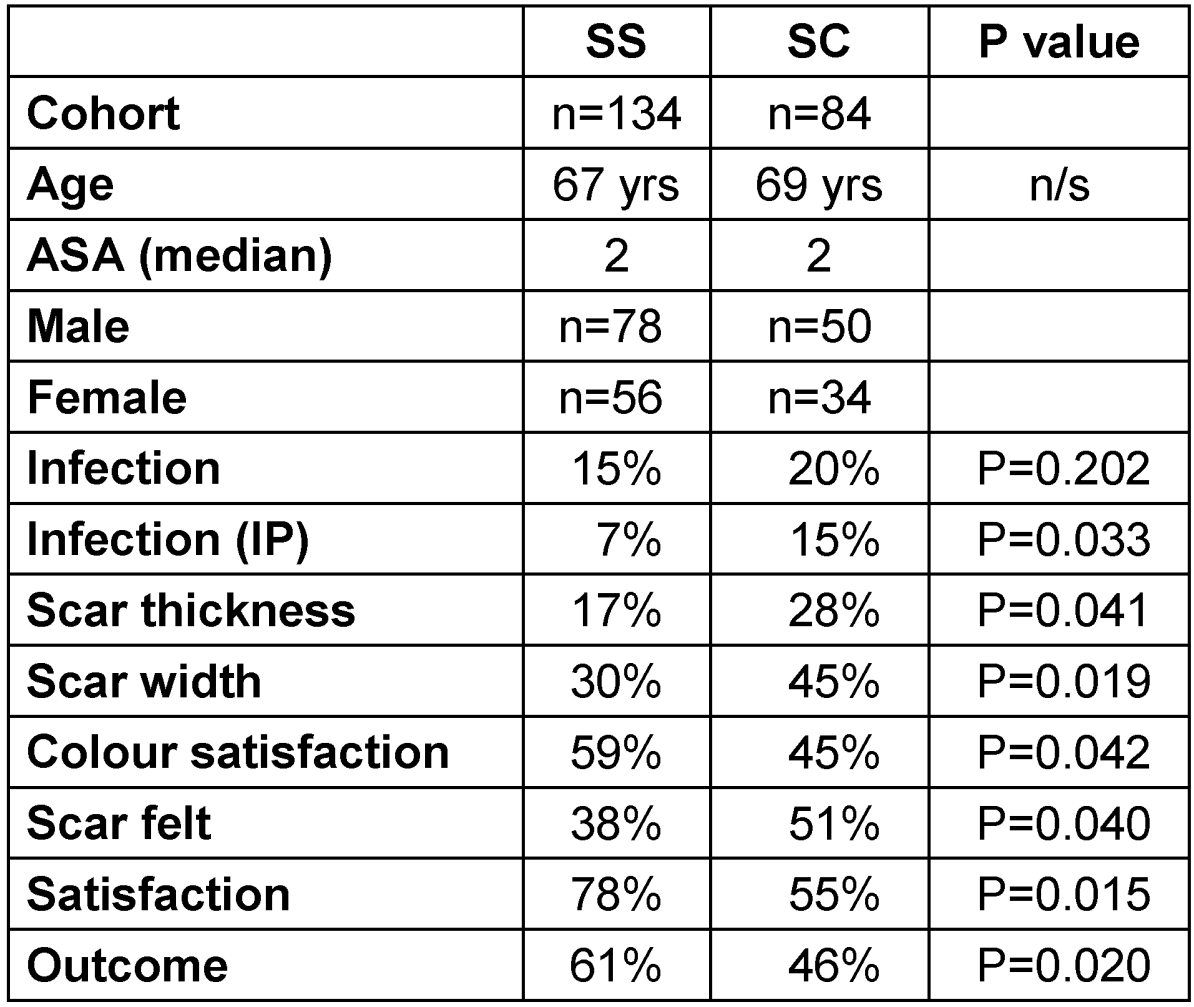
Comparison of results between the SS and SC groups
